# Surgical stress response and long-term survival in robot-assisted versus laparoscopic surgery for colon cancer: a propensity matched nationwide cohort study

**DOI:** 10.1007/s10151-025-03146-y

**Published:** 2025-05-18

**Authors:** Pedja Cuk, A. W. Rosen, M. Mashkoor, M. B. Ellebæk, I. Gögenur

**Affiliations:** 1 Surgical Department, Odense University Hospital, Svendborg, Denmark; 2 Department of Clinical Research, University of Southern Denmark, Odense, Denmark; 3 Department of Surgery, Center for Surgical Science, Zealand University Hospital, Køge, Denmark; 4 Research Unit of Surgery, Odense University Hospital, Odense, Denmark

**Keywords:** Surgical stress response, Robot-assisted surgery, Laparoscopic surgery, Minimally invasive surgery, Colon cancer, Long-term survival, Recurrence

## Abstract

**Purpose:**

This study investigates the potential correlation between the surgical stress response and long-term survival in patients undergoing treatment for colon cancer using either RAS (robot-assisted surgery) or LAS (laparoscopic surgery) and whether this correlation is influenced by the surgical approach. The primary objective was to assess the association between postoperative C-reactive protein (CRP) response and recurrence-free survival in RAS compared with LAS. Secondary endpoints included all-cause mortality and time-to-recurrence.

**Methods:**

This Danish nationwide cohort study included patients diagnosed with Union for International Cancer Control (UICC) stage I–III colon cancer who underwent either RAS or LAS between 2010 and 2018. We employed the Cox proportional regression model to analyze the time-to-event outcomes for both primary and secondary endpoints in patients exhibiting either a low postoperative CRP response (< 80 mg/L) or a high CRP response (CRP ≥ 80 mg/L).

**Results:**

A total of 3484 patients were included in the study, with 490 (14.1%) undergoing RAS and 2994 (85.9%) undergoing LAS. The median follow-up time was 32.5 months (interquartile range [IQR] = 21.0–48.7) for the RAS group and 35.4 months (IQR = 22.8–50.9) for the LAS group. In the RAS group, a lower CRP response (CRP < 80 mg/L) was not associated with improved recurrence-free survival (HR = 0.78, 95% confidence interval [CI] [0.53–1.13], *p* = 0.184), all-cause mortality (hazard ratio [HR] = 0.76, 95% CI [0.46–1.26], *p* = 0.282), or time-to-recurrence (HR = 0.64, 95% CI [0.49–1.06], *p* = 0.079).

**Conclusions:**

The postoperative CRP response was not significantly associated with improved long-term survival outcomes in patients undergoing RAS or LAS for UICC stage I–III colon cancer.

## Introduction

Robot-assisted surgery is increasingly implemented in Denmark for the resection of colon cancer and steadily replaces conventional LAS [1]. It is associated with improved ergonomics, a better field of view [2], and reduced perioperatively induced surgical stress response [3]. The short-term related benefits of RAS include enhanced recovery and lower conversion rates [4, 5]. The influence of the surgical stress response on long-term survival in minimally invasive colon cancer surgery has not been sufficiently studied. However, a nationwide cohort study indicated improved long-term survival using RAS compared with LAS in patients with colon cancer [6].

Even though oncological treatment has advanced in recent decades, surgical treatment remains the cornerstone for resectable colon cancer. However, the degree of intraoperatively induced surgical trauma may be associated with a potential progression of the existing micrometastatic foci through the release of stress hormones, proinflammatory cytokines, and changes in the cellular immune response [7, 8]. The degree of the perioperatively induced systemic inflammation can be monitored by several biomarkers, e.g., neutrophil–lymphocyte ratio and Glasgow Prognostic Score, to predict long-term survival [9, 10]. C-reactive protein, a frequently used acute-phase reactant in the postoperative monitoring of patients undergoing colorectal cancer surgery, can be used in the prediction of long-term survival, as elevated CRP levels in the postoperative course are associated with overall decreased survival rates [11]. This is unequivocal, as a higher CRP response favors overall survival, colorectal-specific survival, and recurrence-free survival. A pronounced CRP response has been shown to correlate with a diminished overall- and colorectal-specific survival, as well as recurrence-free survival in patients undergoing colorectal cancer surgery [11].

The influence of the surgical stress response on long-term survival in patients undergoing RAS for colon cancer has not been studied earlier. This study aims to evaluate a possible association between the perioperatively induced surgical trauma measured by the CRP concentration in the early postoperative course and long-term oncological outcomes in patients undergoing RAS or LAS for UICC stage I–III colon cancer.

## Methods

### Study design, setting, and participants

This observational cohort study examined a possible association between the surgically induced stress response in patients undergoing either RAS or LAS for nonmetastatic colon cancer between 1 January 2010 and 31 December 2018. The cohort included adults (age > 18 years) scheduled for intended curative right, transverse, left-sided, and sigmoid colon resections. Patients undergoing planned surgery for UICC stage I–III colon cancer were identified during the specified period through prospective registration of data recorded in the Danish Colorectal Cancer Group Database (DCCG), thereby excluding patients undergoing emergency surgery. The study was conducted and reported according to the recommendations outlined in The Strengthening the Reporting of Observational Studies in Epidemiology (STROBE) statement: guidelines for reporting observational studies [12].

### Data sources

Data, including the patient’s baseline and peri- and postoperative clinical characteristics, were obtained from the DCCG registry database. It contains information regarding the diagnosis and surgical treatment of adults aged > 18 years undergoing colorectal cancer surgery at Danish public institutions since May 2001. The DCCG has a high accuracy and completeness rate of > 99% [13]. Since all inhabitants of Denmark are provided with a unique security number (CPR) at birth, all records related to health information, including (1) clinical contacts, (2) treatments, (3) cancer registry, and (4) clinical laboratory information, can be linked to the respective CPR number. The Danish National Patient Registry (DNPR) monitors all in- and outpatient admissions, diagnoses specified by the International Classification of Diseases (ICD 8–10), and treatments, including surgical procedures, other interventional treatments, and examinations. It has complete coverage reported from 1978 and is updated continuously [14]. Data related to the diagnosis of malignant tumors, staging, morphology, and grading were collected from the Danish Cancer Registry (DCR) [15]. Information concerning the recurrence of colon cancer was estimated using data from the DCCG registry enriched with the Lash et al. algorithm [16]. Data regarding the postoperative CRP response was collected from RLRR (Register of Laboratory Results for Research).

### Outcomes

The main objective was to evaluate the risk of cancer recurrence among patients who underwent surgery for UICC stage I–III colon cancer, comparing those treated with either RAS or LAS techniques, and to investigate whether this risk was associated with the level of postoperative CRP response. Secondarily, we wanted to examine the association between (1) all-cause mortality, (2) time-to-recurrence, and (3) the postoperative CRP response. Recurrence was defined as indicated by Lash et al. in a validated algorithm collecting data from several Danish health registers (DNPR, DPR, and DCR). Local or distant recurrence was identified searching the DNPR in case of a registered: (1) metastasis code (ICD 76–80) after 180 days following the primary surgical procedure and without evidence of a newly registered cancer diagnosis; (2) cytostatic therapy code (BWHA 1–2, BOHJ17 of BOHJ19B1) 180 days after the surgical procedure or 60 days following the last cytostatic therapy, and without having a new cancer; (3) DPR registered Systematized Nomenclature of Medicine—Clinical Terms (SNOMED) codes 180 days or more after index surgery; or (4) a specific code consistent with local recurrence registered in the DNPR. According to the algorithm, patients were excluded if colorectal cancer or recurrence diagnosis was registered within 180 days after the primary diagnosis, except individuals diagnosed with nonmelanoma skin cancer [16]. The 180-day limit was applied to both primary and secondary outcomes, thereby excluding patients with evidence of recurrent disease.

### Statistics

**Fig. 1:**
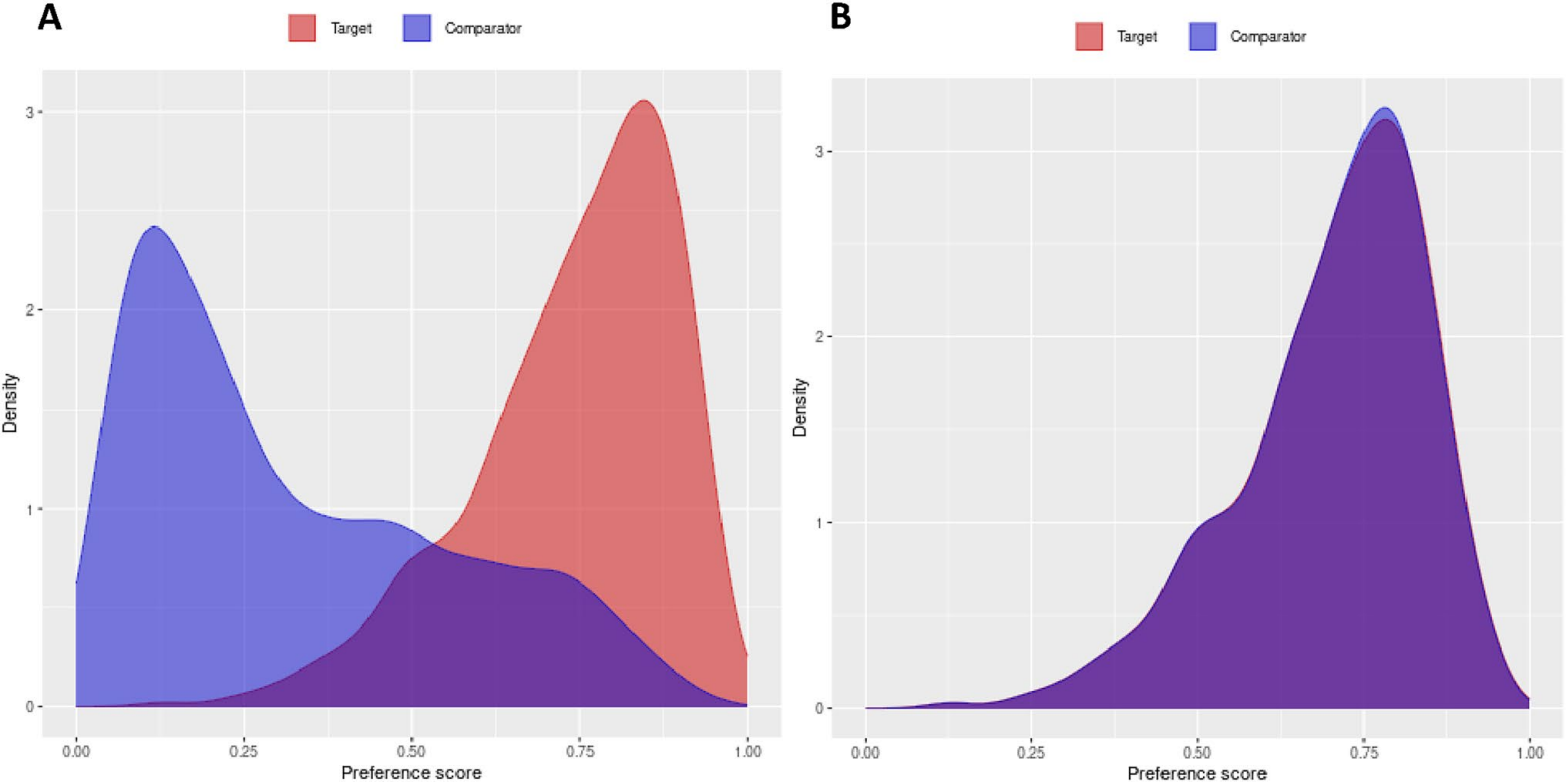
Density plot of propensity scores before (**A**) and after (**B**) matching in patients undergoing RAS (target) and LAS (comparator) for UICC stage I–III colon cancer

Data were extracted from DCCG, Clinical Laboratory Register, and DNPR data and transformed to The Observational Medical Outcomes Partnership Common Data Model (OMOP-CDM)[17]. Propensity scores (PS) were estimated using a LASSO logistic regression, which conducts a penalized likelihood regression [18, 19]. Covariates included in the PS model were related to the respective group’s demographic characteristics, measurements, and ICD diagnosis and procedure codes. Sensitivity analyses were conducted using PS matching to assess whether the reported estimates were affected by baseline confounding and thereby the degree of treatment assignment bias by matching exposed with nonexposed individuals. Patients were matched in a 1:1 ratio upon their estimated PS with a max calipher width of 0.2 on the logit scale as proposed by Austin et al. [20, 21]. A Cox proportional hazard regression model was used to verify any potential effect modification of the postoperative CRP response and long-term survival, stratified by a low postoperative CRP response (< 80 mg/L) compared with the group with a high CRP response (≥ 80 mg/L) across postoperative day 1–3. The assumptions underlying this cutoff threshold stem from data obtained in an observational study comparing patients undergoing planned RAS versus LAS for colorectal cancer [22]. Results of both the unadjusted and adjusted analyses following PS matching are shown in Fig. 1. Patients with missing data, including covariates incorporated in the PS model, were excluded from the Cox proportional hazard regression analysis. We did not perform a prior sample size calculation for the main objective because there was no existing reporting on whether the degree of CRP response was associated with long-term survival and the choice of surgical method. A two-sided *p*-value < 0.05 was considered statistically significant. Cohorts were identified using the ATLAS platform (version 2.7.3), and the statistical analyses were carried out in RStudio (version 4.2.0) using the cohort method package (version 4.2.2).

## Results

A total of 3484 patients, 490 (14.1%) undergoing RAS and 2994 (85.9%) undergoing LAS, were identified from January 2010 through December 2018 before PS matching, leaving 384 patients after PS matching in each study group. The median follow-up time in the groups was {RAS = 32.5 months (IQR = 21.0–48.7) and LAS = 35.4 months (IQR = 22.8–50.9)}, respectively. The mean age of patients included did not differ between the two groups {RAS = 70.6 years (standard deviation [SD] 10.1) and LAS = 70.3 years [SD 10.1]}, Table 1. Most of the cohort consisted of patients without significant prior comorbidities (American Society of Anesthesiologists [ASA] 1–2, World Health Organization [WHO] performance status 0–1, and Charlson Comorbidity Index 0–1) (Table 1). The cohort consisted predominantly of patients diagnosed in UICC stage III and did not differ between the two study groups (RAS = 114 [23.3%]; LAS = 646 [21.6%]). However, there was a considerable rate of missing data regarding the clinical UICC stage (RAS = 246 [50.2%]; LAS = 1536 [51.3%]). A higher postoperative CRP concentration (CRP ≥ 80 mg/L) was to a greater extent present in both study groups across all measurements (RAS = 293 [59.8%]; LAS = 1796 [60.0%]).

**Table 1:** Baseline characteristics

Variable	Before PS matching			After PS matching		
	RAS (490)	LAS (2994)	SMD	RAS (384)	LAS (384)	SMD
Age, years (mean, SD)	70.6 (10.1)	70.3 (10.1)	0.03	70.1 (9.9)	70.3 (9.9)	0.02
BMI						
≤ 18.5	13 (2.7)	70 (2.3)	0.02	10 (2.6)	9 (2.3)	0.02
> 18.5 and ≤ 25	178 (36.3)	1107 (40.0)	0.01	139 (36.2)	141 (36.7)	0.02
> 25 and ≤ 30	173 (35.3)	1084 (36.2)	0.02	132 (34.4)	148 (38.5)	0.08
> 30 and ≤ 35	75 (15.3)	467 (15.6)	0.01	61 (15.9)	53 (13.8)	0.07
> 35	37 (7.6)	182 (6.1)	0.06	29 (7.6)	22 (5.7)	0.04
Missing	14 (2.9)	84 (2.8)	NA	13 (3.4)	11 (2.9)	NA
Gender						
Female	210 (42.9)	1421 (47.5)	0.09	173 (45.1)	170 (44.3)	0.02
Male	280 (57.1)	1573 (52.3)	0.09	211 (54.9)	214 (55.7)	0.02
Tumor location						
Cecum	67 (13.7)	506 (16.9)	0.08	52 (13.5)	57 (14.8)	0.03
Ascending colon	132 (26.9)	668 (22.3)	0.10	101 (26.3)	87 (22.7)	0.08
Transverse colon	25 (5.1)	196 (6.5)	0.06	21 (5.4)	22 (5.7)	0.01
Descending colon	40 (8.2)	261 (8.7)	0.01	30 (7.8)	26 (6.8)	0.04
Sigmoid colon	223 (45.6)	1356 (45.3)	0.00	178 (46.3)	190 (49.8)	0.0
Missing	< 6 (0)	7 (0.2)	–	< 6 (0)	< 6 (0)	–
ASA-score						
ASA 1	118 (24.1)	720 (24.0)	0.00	87 (22.7)	89 (23.2)	0.01
ASA 2	274 (55.9)	1645 (54.9)	0.01	219 (57.0)	211 (54.9)	0.04
ASA 3	88 (18.0)	588 (19.6)	0.04	71 (18.5)	81 (21.1)	0.06
ASA 4	6 (1.2)	19 (0.6)	0.06	< 6 (0)	<6 (0)	0.08
Missing	< 6 (0)	22 (0.7)	–	< 6 (0)	<6 (0)	–
UICC stage						
UICC 1	81 (16.5)	513 (17.1)	0.02	62 (16.1)	64 (16.7)	0.01
UICC 2	49 (10.0)	299 (10.0)	0.00	40 (14.1)	37 (9.6)	0.03
UICC 3	114 (23.3)	646 (21.6)	0.04	94 (24.5)	88 (22.9)	0.04
Missing	246 (50.2)	1536 (51.3)	–	188 (49.0)	195 (50.8)	–
Performance status						
0	301 (61.4)	1859 (62.1)	0.01	236 (61.5)	248 (64.6)	0.06
1	104 (21.2)	601 (20.1)	0.03	78 (20.3)	71 (18.5)	0.05
2	34 (6.9)	166 (5.5)	0.06	29 (7.6)	20 (5.2)	0.10
3	8 (1.6)	31 (1.0)	0.05	6 (1.6)	< 6 (0)	0.07
4	< 6 (0)	6 (0.2)	–	< 6 (0)	NR	–
Missing	39 (8.0)	331 (11.1)	–	30 (7.8)	42 (10.9)	–
Alcohol consumption						
0 units (week)	96 (19.6)	593 (19.8)	0.00	74 (19.3)	75 (19.5)	0.00
1–14 units (week)	315 (64.3)	1838 (61.4)	0.06	246 (64.1)	242 (63.0)	0.02
15–21 units (week)	24 (4.9)	232 (7.7)	0.11	20 (5.2)	33 (8.6)	0.13
> 21 units (week)	19 (3.9)	162 (5.4)	0.07	16 (4.2)	22 (5.7)	0.07
Missing	36 (7.3)	169 (5.6)	–	28 (7.3)	12 (3.1)	–
Charlson comorbidity index						
0	321 (65.5)	1876 (62.7)	0.06	248 (64.6)	243 (63.3)	0.02
1	99 (20.2)	662 (22.1)	0.05	79 (20.6)	82 (21.4)	0.02
2	39 (10.2)	266 (8.9)	0.05	32 (8.3)	33 (8.6)	0.01
3	31 (6.3)	189 (6.3)	0.00	25 (6.5)	26 (6.8)	0.01
Missing	0 (0)	< 6 (0)	–	0 (0)	0 (0)	–
CRP (mg/L)						
CRP < 80	197 (40.2)	1198 (40.0)	0.00	159 (41.4)	167 (43.5)	0.04
CRP ≥ 80	293 (59.8)	1796 (60.0)	0.00	225 (58.6)	217 (56.5)	0.04

*BMI* body mass index, *ASA* American Society of Anesthesiologists, *UICC* Union for International Cancer Control, *CRP*   C-reactive protein, *RAS* robot-assisted surgery, *LAS*   laparoscopic surgery, *PS* propensity score, *SMD*  standard mean difference

### Primary outcome—recurrence free survival

**Fig. 2:**
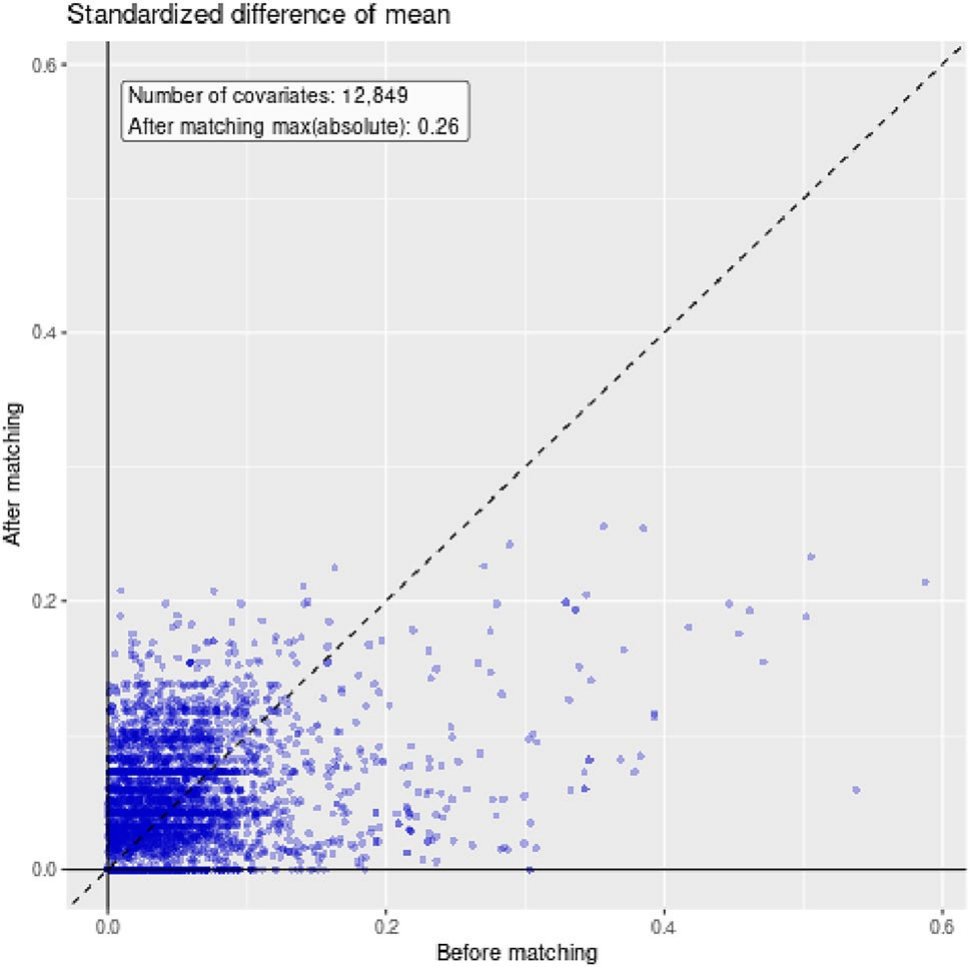
Scatter plot of covariate balance in patients undergoing RAS or LAS for UICC stage I–III colon cancer

Regarding the primary outcomes (recurrence-free survival), a low postoperative CRP response < 80 mg/L if undergoing RAS did not cause a lower risk of recurrence (HR_unadjusted_ = 0.78, 95% CI [0.53–1.13], *p* = 0.184), (HR_adjusted_ = 0.50, 95% CI [0.22–1.15], *p* = 0.102). According to the density plot, the propensity scores were equally distributed after PS matching (Fig. 1b*)*. A total of 0.25% of covariates were above the threshold of 0.1 following PS matching (Fig. 2*).* A high CRP response ≥ 80 mg/L was not associated with an increased risk of recurrence-free survival if undergoing RAS (HR_unadjusted_ = 1.09, 95% CI [0.83–1.44], *p* = 0.540; HR_adjusted_ = 1.54, 95% CI [0.84–2.82], *p* = 0.166; Table 2). The 3-year risk of recurrence was 11.3% (50/489) in the RAS and 14.8% (386/2993) in the LAS group.

**Table 2:** Survival analysis, including **a** recurrence-free survival, *b* time-to-recurrence, and *c* overall survival in patients undergoing RAS or LAS for UICC stage I–III colon cancer.stratified according to the postoperative C-reactive protein (CRP) response (low CRP response group = CRP_RAS_ < 80 mg/L versus high CRP response group = CRP_RAS_ ≥ 80 mg/L)

Variable	CRP_RAS_ < 80 mg/L, 95 % CI	*p*	CRP_RAS_ ≥ 80 mg/L, 95 % CI	*p*
Recurrence-free survival	*LAS = ref*		*LAS = ref*	
	HR_unadjusted_ = 0.78, [0.53–1.13]	0.184	HR_unadjusted_ = 1.09, [0.83–1.44]	0.540
	HR_adjusted_ = 0.50, [0.22–1.15]	0.102	HR_adjusted_ = 1.54, [0.84–2.82]	0.166
Time-to-recurrence	*LAS = ref*		*LAS = ref*	
	HR_unadjusted_ = 0.64, [0.49–1.06]	0.079	HR_unadjusted_ = 1.05, [0.73–1.51]	0.808
	HR_adjusted_ = 0.45, [0.12–1.73]	0.247	HR_adjusted_ = 1.15, [0.49–2.72]	0.743
All-cause mortality	*LAS = ref*		*LAS = ref*	
	HR_unadjusted_ = 0.76, [0.46–1.26]	0.282	HR_unadjusted_ = 1.13, [0.80–1.60]	0.491
	HR_adjusted_ = 0.71, [0.26–1.89]	0.247	HR_adjusted_ = 1.63, [0.80–3.30]	0.176

*RAS* robot-assisted surgery, *LAS* laparoscopic surgery, *Ref* Reference, *UICC*  Union for International Cancer Control

### Secondary outcomes

The 3-year all-cause mortality rate was the following in the two groups (RAS = 11.0% [54/489]; LAS = 11.9% [356/2993]). The surgically induced stress response was not associated with the all-cause mortality rate in either the group with the low CRP response < 80 mg/L (HR_unadjusted_ = 0.76, 95% CI [0.46–1.26], *p* = 0.282; HR_adjusted_ = 0.71, 95% CI [0.26–1.89], *p* = 0.247) or the group with the high CRP response in case of  CRP response ≥ 80 mg/L (HR_unadjusted_ = 1.13, 95% CI [0.80–1.60], *p* = 0.491; HR_adjusted_ = 1.63, 95% CI [0.80–3.30], *p* = 0.176) (Table 2).

The 3-year disease-free survival rate was 17.8% in the RAS and 20.2% in the LAS group, respectively. The postoperative CRP concentration was not associated with a statistically significant reduction in time-to-recurrence in case of either having a low postoperative CRP response < 80 mg/L (HR_unadjusted_ = 0.64, 95% CI [0.49–1.06], *p* = 0.079; HR_adjusted_ = 0.45, 95% CI [0.12–1.73], *p* = 0.247) or higher inflammatory response in case of CRP response ≥ 80 mg/L (HR_unadjusted_ = 1.05, 95% CI [0.73–1.51], *p* = 0.808; HR_adjusted_ = 1.15, 95% CI [0.49–2.72], *p* = 0.743) (Table 2).

## Discussion

This nationwide cohort study examined a possible association between the surgically induced trauma expressed by the postoperative CRP response and long-term survival in patients undergoing RAS or LAS for UICC stage I–III colon cancer. No statistically significant differences could be detected in either recurrence-free survival, all-cause mortality, or time-to-recurrence between the study groups depending on level of CRP response in the early postoperative course.

Robot-assisted surgery is a well-established surgical technique for rectal cancer. However, the evidence for its systemic application for colon cancer is still limited, and LAS is still practiced to a greater extent. Registry-based cohort studies have been published examining the long-term outcomes of RAS and LAS for colon cancer [6, 23, 24]. Mirkin et al. demonstrated a significantly improved overall survival rate in UICC stage II and III colon cancer in favor of RAS. To our knowledge, no studies, including larger sample sizes, have examined the association between the surgical stress response and long-term survival induced by RAS or LAS. A retrospective cohort study including 298 patients undergoing RAS or LAS for colorectal cancer in a planned setting indicated that LAS was associated with a significantly increased CRP response in the early postoperative course [22].

A higher CRP response following surgery is associated with an increased risk of cancer recurrence, all-cause mortality, and time-to-recurrence in patients with colorectal cancer [25, 26]. A possible explanation is that perioperatively induced surgical trauma is directly correlated with the risk of residual disease and recurrent cancer caused by either local colonization of residual tumor cells or transit into lymphatic- or bloodstream-causing micrometastases [27]. Few studies reported the significance of a postoperatively elevated CRP response as a marker for long-term survival in patients undergoing minimally invasive surgery for colorectal cancer. However, more recently published studies found a correlation between a high postoperative inflammatory response (CRP and interleukin-6) and worsened long-term survival in patients undergoing LAS and open surgery for UICC stage I–III colorectal cancer [26, 28]. The following risk factors were associated with a higher risk of recurrent disease: increasing age and BMI, right-sided colon cancer, male gender, and extensive surgical time consumption [29]. Despite the study not demonstrating any statistically significant association between long-term survival and the postoperative CRP response depending on the choice of surgical method, there was a certain association, as a lower CRP response < 80 mg/L had a protective effect on long-term survival. The lack of demonstrating this association could possibly be owing to underpowering, as the study did not include a high number of robot-assisted cases. Recent studies have shown a clear association between a higher postoperative CRP response and poorer overall and colorectal-specific survival [11, 30]. A longer inclusion period with a higher proportion of robot-assisted cases would clarify whether there is a statistically significant association between surgically induced trauma and long-term survival. Nonetheless, the existing results can be used as an indicator that the use of RAS is associated with reduced surgical trauma in the early postoperative phase, which may improve recurrence-free survival. Clinically, these results could facilitate more personalized medicine during the perioperative period by identifying patients at higher risk through regular monitoring of inflammatory markers, such as CRP. These markers may be targeted for suppression using immunomodulatory agents. Furthermore, the inflammatory response, as indicated by these selected markers, can be utilized to monitor patients in the postoperative phase receiving immunotherapy, enabling individual and tailored treatment.

The main limitations of the present study primarily relate to unmeasured confounding due to non-balancing of potential covariates in the propensity score matched model. There may exist a systematic selection of patients with higher BMI (≥ 30 kg/m^2^) in case of higher UICC stage (UICC ≥ II) and with a worse performance status (≥ 1) in the RAS group. This selection could result in a higher postoperative complication rate and impaired long-term survival [31, 32]. Owing to a high proportion of missing data regarding the UICC stage in the two surgical modalities, it is possible that this contributes to a higher degree of confounding, which can cause bias in the point estimates. However, the distribution of missing data is random/equally distributed between the groups, so the degree of selection bias is limited. As the Lash algorithm of cancer recurrence excludes patients having recurrent disease within 180 days after primary surgery, the generalizability of the results can be discussed since the time-at-risk does not represent the entire inclusion period. Moreover, the study is predominantly limited by underpowering owing to a presumed lack of robot-assisted cases, which, in comparison with laparoscopic procedures, has been practiced to a lesser extent. Given the lack of previous studies examining the association between RAS versus LAS, the inflammatory stress response (as indicated by CRP levels), and long-term survival in patients who underwent colon cancer surgery, an arbitrary CRP cutoff point was established for this analysis. This approach is supported by a retrospective study [22] that previously investigated the impact of RAS versus LAS on colorectal cancer surgery outcomes. To ensure the robustness and reliability of our findings, additional sensitivity analyses were conducted to assess any potential effect modification of the CRP response on long-term survival. However, the strengths of this study can be attributed to the national Danish registries with a high completeness rate; likewise, a uniform public health system minimizes the risk of patient selection by minimizing the impact of socioeconomic factors. Furthermore, data-driven large-scale propensity matching, as applied in our study, can minimize the degree of confounding by including a high amount of covariates in the propensity score model [33]. Merging data from public health registries in combination with the clinical colorectal database (DCCG) ensures a more detailed cohort description [13].

In conclusion, the degree of postoperative CRP response was not associated with improved long-term survival outcomes in patients undergoing RAS or LAS for UICC stage I–III colon cancer.

## Data Availability

No datasets were generated or analyzed during the current study.
